# Evaluation of whole-genome DNA methylation sequencing library preparation protocols

**DOI:** 10.1186/s13072-021-00401-y

**Published:** 2021-06-19

**Authors:** Jacob Morrison, Julie M. Koeman, Benjamin K. Johnson, Kelly K. Foy, Ian Beddows, Wanding Zhou, David W. Chesla, Larissa L. Rossell, Emily J. Siegwald, Marie Adams, Hui Shen

**Affiliations:** 1 Department of Epigenetics, Van Andel Research Institute, 333 Bostwick Avenue NE, Grand Rapids, MI 49503 USA; 2 Genomics Core, Van Andel Research Institute, 333 Bostwick Avenue NE, Grand Rapids, MI 49503 USA; 3 Center for Computational and Genomic Medicine, The Children’s Hospital of Philadelphia, 3501 Civic Center Boulevard, Philadelphia, PA 19104 USA; 4 Department of Pathology and Laboratory Medicine, University of Pennsylvania, Philadelphia, PA 19104 USA; 5 Spectrum Health Office of Research and Education, Spectrum Health System, 15 Michigan Street NE, Grand Rapids, MI 49503 USA

**Keywords:** DNA methylation, Epigenetics, Whole-genome bisulfite sequencing, Enzymatic methylation sequencing, Fallopian tube

## Abstract

**Background:**

With rapidly dropping sequencing cost, the popularity of whole-genome DNA methylation sequencing has been on the rise. Multiple library preparation protocols currently exist. We have performed 22 whole-genome DNA methylation sequencing experiments on snap frozen human samples, and extensively benchmarked common library preparation protocols for whole-genome DNA methylation sequencing, including three traditional bisulfite-based protocols and a new enzyme-based protocol. In addition, different input DNA quantities were compared for two kits compatible with a reduced starting quantity. In addition, we also present bioinformatic analysis pipelines for sequencing data from each of these library types.

**Results:**

An assortment of metrics were collected for each kit, including raw read statistics, library quality and uniformity metrics, cytosine retention, and CpG beta value consistency between technical replicates. Overall, the NEBNext Enzymatic Methyl-seq and Swift Accel-NGS Methyl-Seq kits performed quantitatively better than the other two protocols. In addition, the NEB and Swift kits performed well at low-input amounts, validating their utility in applications where DNA is the limiting factor.

**Results:**

The NEBNext Enzymatic Methyl-seq kit appeared to be the best option for whole-genome DNA methylation sequencing of high-quality DNA, closely followed by the Swift kit, which potentially works better for degraded samples. Further, a general bioinformatic pipeline is applicable across the four protocols, with the exception of extra trimming needed for the Swift Biosciences’s Accel-NGS Methyl-Seq protocol to remove the Adaptase sequence.

**Supplementary Information:**

The online version contains supplementary material available at 10.1186/s13072-021-00401-y.

## Background

The dynamic interplay between various epigenetic modifications influence cellular differentiation, lineage specification, tissue development, and can also promote oncogenic states through changes in histone modifications and DNA methylation. In mammals, DNA methylation typically occurs at the 5’ position of CpG dinucleotides (denoted 5-mC) and remains the single best studied epigenetic mark, partly due to its robustness through most storage conditions and histological preparations, such as formalin-fixed, paraffin-embedded (FFPE) samples [1].

DNA methylation can be assayed with various principles [2], such as bisulfite conversion [3], restriction enzyme digestion, or differential affinity for methylated DNA binding proteins. Array-based and sequencing-based methods built upon these principles have been developed and benchmarked [4–6]. The current gold-standard approach to examine genome-wide DNA methylation composition and differences is through chemical modification of unmethylated cytosines using sodium bisulfite [7]. Bisulfite deaminates unmethylated cytosines (Cs) to uracils that are converted to thymines (Ts) during PCR amplification. Methylated cytosines (mCs) remain unaltered through this process. The end result yields stable genetic differences between methylated (C) and unmethylated cytosines (T), reflecting the underlying DNA methylation landscape, effectively turning the epigenetic difference into a genetic difference, which can then be studied using conventional genome-scale methods, such as microarrays or sequencing. Various generations of bisulfite-based microarrays have been used to profile hundreds of thousands of human samples due to the low cost and easy, standardized data processing and analysis. With dropping sequencing cost in recent years, the popularity of sequencing-based methods has been on the rise [8].

Whole-genome bisulfite sequencing (WGBS) provides the most comprehensive single base resolution DNA methylation maps. It was successfully applied to *Arabidopsis thaliana* in 2008 [9, 10] and then to humans in 2009 [11]. In these early methods, adapter-ligated library material undergoes bisulfite conversion, leading to sheared and degraded library fragments and overall lower quantities and diversity of sequenceable material. A post-bisulfite adapter tagging (PBAT) method [12, 13] was developed to overcome this hurdle, effectively decreasing the input range to nanogram level. Notably, this method has been used for single-cell WGBS profiling [14]. More recently, Swift Biosciences has produced a kit that is an alternative approach to the post-bisulfite library preparation. The alternative approach maintains the low DNA input capabilities of PBAT, while also including a low-complexity sequence on the 3′ end of the ssDNA during library preparation that serves as a scaffold for sequencing adapter attachment (Accel-NGS Methyl-Seq protocol, Swift Biosciences).

The conditions needed for bisulfite conversion are known to be harsh on the DNA and cause degradation. In recent years, it has become clear that this conversion can also be achieved with an enzymatic approach. 5-mCs can be converted to 5-hydroxymethylcytosine (5-hmC), then to 5-formylcytosine (5-fC), and eventually to 5-carboxylcytosine (5-caC) by the ten–eleven translocation (TET) family dioxygenases [15]. Further, the apolipoprotein B mRNA editing enzyme, catalytic polypeptide-like 3A (APOBEC3A) deaminates methylated and unmethylated Cs into thymines and uracils, respectively [15]. While both 5-mCs and Cs are affected by this process, the TET-oxidized methylcytosines, 5-hmC, 5-fC, and 5-caC are minimally impacted [15]. Based on this principle, an enzymatic methyl-seq (EM-seq) method was recently developed by New England Biolabs. Their method uses TET2 to oxidize methylated cytosines and subsequent APOBEC3A treatment to convert unmethylated cytosines to uracils [15]. WGBS and EM-seq, collectively referred to as whole-genome methylation sequencing (WGMS), both convert a 5-mC/C difference to a C/T difference; therefore, analysis tools developed for WGBS are also applicable to EM-seq.

In this study, we extensively benchmark the performance of three most commonly used protocols for bisulfite-based whole-genome DNA methylation profiling including the KAPA Hyper Prep kit (Kapa), the Miura and Ito post-bisulfite adapter tagging (PBAT) method, and the Swift Biosciences Accel-NGS Methyl-Seq DNA library kit (Swift), as well as the new EM-seq protocol from New England Biolabs, the NEBNext Enzymatic Methyl-seq kit (NEB), on fresh–frozen human tissue samples. For each technique, we evaluate input quantities, read mapping statistics, library complexity, insert size, cytosine retention, as well as reproducibility between replicates. We also present bioinformatic analysis pipelines for each of these library types.

## Results

Benchmarking studies often use cell lines due to the largely isogenic background and reduced biological variance to probe the reproducibility of the method. However, it is often of interest to apply these approaches to complex tissue sources for primary research. Thus, we chose to use frozen normal primary human solid tissue to benchmark these kits in a more common research scenario. We used human fallopian tube samples, which are believed to host the presumed cell-of-origin for high-grade serous ovarian cancer [16]. The results presented are based on two snap-frozen primary patient fallopian tube samples (denoted Biological Replicates A and B). DNA from each sample was prepared using one of the four library preparation protocols, as summarized in Table 1. With the exception of the PBAT protocol, two aliquots from the same DNA extraction were used to produce technical replicates for each protocol (denoted Technical Replicates 1 and 2). The PBAT protocol does not have a technical replicate due to the poor quality of the initial sequencing run and not enough leftover library material to generate additional sequencing information for either replicate. In addition to generating samples using the suggested amount of DNA input, a smaller DNA input (10 ng each) was used in the NEB and Swift protocols to test the effectiveness of low DNA inputs on these protocols, which performed best in the initial testing. (NEB and Swift state they can go as low as 10 ng [17] and 100 pg [18], respectively.) Table 1 shows the details for each protocol used in this study.

**Table 1:** Summary of library preparation protocols used in analysis

Short Name	Kapa	NEB	PBAT	Swift	Low NEB	Low Swift
Company Name	Roche Sequencing	New England Biolabs	N/A	Swift Biosciences	New England Biolabs	Swift Biosciences
Kit / Protocol Name	KAPA Hyper Prep	NEBNext Enzymatic Methyl-seq	Miura F., Ito T. (2018)	Accel-NGS Methyl-Seq	NEBNext Enzymatic Methyl-seq	Accel-NGS Methyl-Seq
Kit or Protocol?	Kit	Kit	Protocol	Kit	Kit	Kit
Strands aligned to	OT/OB	OT/OB	CTOT/CTOB	OT/OB	OT/OB	OT/OB
WGBS or EM-seq?	WGBS	EM-seq	WGBS	WGBS	EM-seq	WGBS
DNA Input [ng]	300	200	100	100	10	10
# Samples	2 (A/B)	2 (A/B)	2 (A/B)	2 (A/B)	2 (A/B)	2 (A/B)
# Tech. Reps.	2 (1/2)	2 (1/2)	1 (1)	2 (1/2)	2 (1/2)	2 (1/2)
Sheared?	Yes	Yes	No	Yes	Yes	Yes
Conversion Kit	EZ DNA Methylation-Gold kit	N/A	EZ DNA Methylation-Gold kit	EZ DNA Methylation-Gold kit	N/A	EZ DNA Methylation-Gold kit
# Amplification Rounds	10	4	0	4	8	8
DNA Controls	lambda phage / pUC19	lambda phage / pUC19	lambda phage / pUC19	lambda phage / pUC19	lambda phage / pUC19	lambda phage / pUC19
Sequencer Used	Illumina NovaSeq6000	Illumina NovaSeq6000	Illumina NovaSeq6000	Illumina NovaSeq6000	Illumina NovaSeq6000	Illumina NovaSeq6000
Approx. Processing Time [hrs]	7	9	14-16	7	9	7

Throughout the text, the “Short Name” entry is used to describe which protocol is being discussed, rather than the full name. Sample and technical replicate names are included in parentheses. The lambda phage control is unmethylated, while the pUC19 control is methylated. These were added to the high molecular weight genomic DNA sample at a rate of 0.01% and 0.0005%, respectively. The OT and OB strands are the original top and original bottom strands, while the CTOT and CTOB are the complements of the original top and original bottom strands, respectively. These strands can also be referred to as the bisulfite Watson (OT), bisulfite Crick (OB), bisulfite Watson reverse (CTOT), and bisulfite Crick reverse (CTOB) strands

**Fig. 1:**
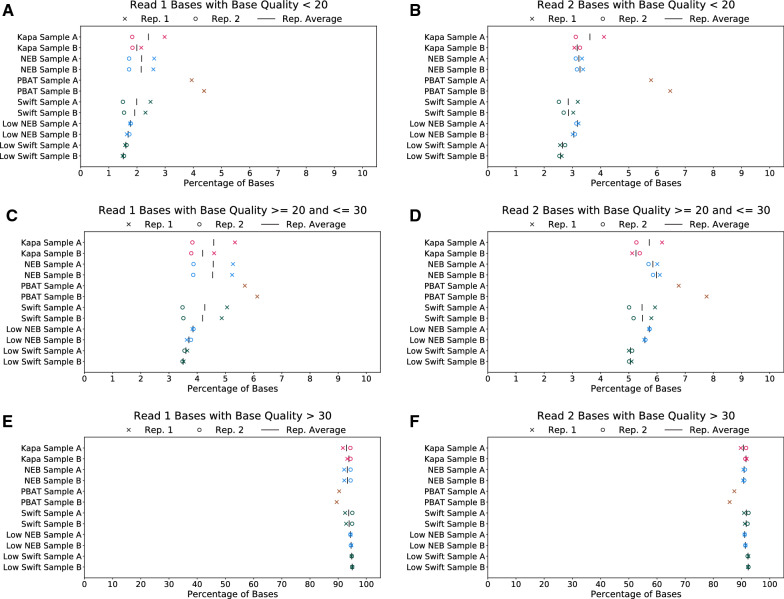
Raw read statistics for each protocol. Each plot shows the percentage of bases with different levels of base quality, namely low base quality (< 20) for **A** read 1 and **B** read 2, medium base quality ($20 \le$ quality $\le 30$) for **C** read 1 and **D** read 2, and high base quality (> 30) for **E** read 1 and **F** read 2

The first set of metrics compared between the four preparations is related to the quality of raw reads received from the sequencer, including base quality and adapter contamination, and the effects of trimming on those reads. In general, the raw base qualities, percentage of reads with adapter contamination, and percentage of bases trimmed are comparable between the Kapa, NEB, and Swift protocols (Fig. 1 and Additional file 1: Figure S1). However, the PBAT protocol suffers from a higher percentage of low-quality bases along the length of the read, leading to a higher percentage of trimmed bases during the trimming stage. Because the PBAT protocol does not contain an amplification step during library preparation, the higher percentage of trimmed bases relative to the other preparations has a larger effect on the amount of usable data available from this protocol.

**Fig. 2:**
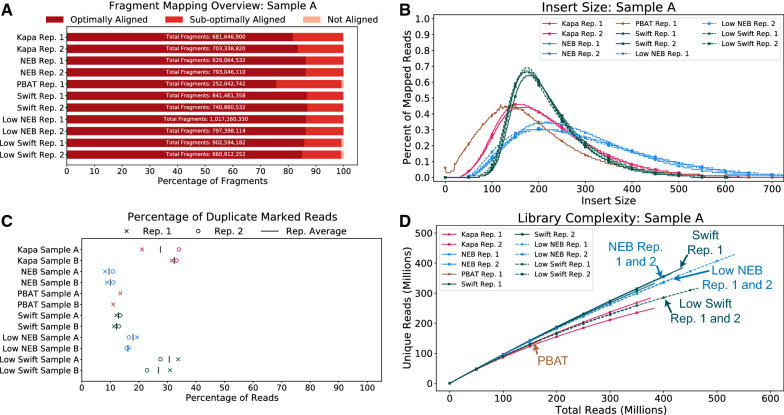
Library quality metrics for each protocol for Sample A. **A** The percentage of optimally, sub-optimally, and not aligned read fragments for each protocol. Note, read fragments treat reads 1 and 2 as separate entities, as it is possible that one read in the pair is mapped, while the other is not. Additional file 1: Figure S2 shows the number of read fragments shown on each bar. **B** Insert size distribution. **C** Duplicate rate for reads with MAPQ $\ge 40$. **D** The library complexity, which is a function of the duplicate rate. Metrics for Sample B are shown in Additional file 1: Figure S3

Following adapter and quality trimming of the raw reads, the reads were mapped to the human genome and the fraction of optimally aligned (defined as MAPQ $\ge 40$), sub-optimally aligned (MAPQ $<40$), and not aligned read fragments was calculated. For Sample A, the NEB and Swift protocols had about the same fraction of read fragments that were optimally aligned ($\sim 85$%), regardless of the amount of input DNA (Fig. 2A). The Kapa protocol was slightly behind ($\sim 80$%), while the PBAT protocol was closer to $\sim 75$%. The lower percentage of optimally aligned reads for the PBAT protocol, when coupled with the substantially lower number of read fragments, means there is much less data to use when performing analyses using this protocol.

Another metric that can speak to the quality of a library preparation is the insert size, which relates to the size of the sequenced DNA fragments. For these experiments, DNA used in the Kapa, NEB and Swift libraries were generated in a single reaction, then split into individual aliquots for library generation. Given the uniformity of input, it would be expected that the resulting libraries would also have identical size profiles. However, bisulfite preparations (Kapa and Swift) led to shorter fragments compared to the enzymatic preparation (NEB), which retained a wider range of fragment lengths that are generally longer than the bisulfite fragments (Fig. 2B). The retention of a smaller range of shorter fragments by the bisulfite preparations suggest degradation of the DNA during library generation, which is consistent with the known tendency of bisulfite conversion to degrade samples. Whereas the other samples were sheared prior to conversion, the PBAT samples were only sheared by the bisulfite process itself. This process is quite destructive, leaving shorter fragments than the other bisulfite conversion protocols. The Swift protocol had the longest and more consistent insert size out of all bisulfite-based methods.

With regards to the fraction of reads with MAPQ $\ge 40$ marked as duplicates (Fig. 2C), the standard-input NEB, PBAT, and Swift protocols each had about 10% of duplicate reads. The low-input NEB protocol was slightly higher at $\sim 15$%. The Kapa (standard-input) and low-input Swift protocols had the highest percentage of reads, sitting closer to 25%.

The library complexity is a metric that can be used to determine if a library has reached a saturation point, where sequencing deeper will only gain a marginal amount of unique (i.e., not duplicate) reads. While none of the samples were sequenced to saturation, the Kapa and low-input Swift samples showed a lower level of complexity compared with the NEB and standard-input Swift samples (Fig. 2D). Due to the PBAT samples having much fewer reads, it is difficult to ascertain where the PBAT library complexity ranks compared to data derived from the NEB or Kapa protocols at higher read depths. At the PBAT sequencing depth in this study, it appears to have a trend similar to the Kapa protocol complexities, implying an overall lower level of library complexity relative to Swift and NEB data.

**Fig. 3:**
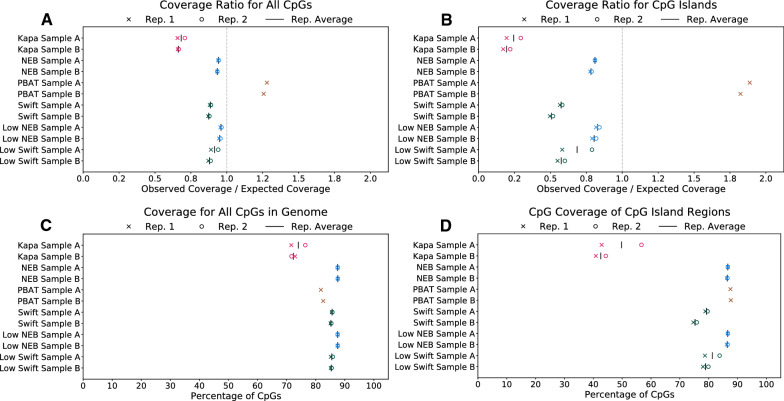
Library uniformity as measured by coverage of various genomic element categories. Ratio of observed coverage to expected coverage for **A** all CpGs and **B** CpG islands. **C** Percentage of all CpGs covered by at least one unique read with MAPQ $\ge 40$. **D** Percentage of CpGs in CpG islands covered by at least one unique read with MAPQ $\ge 40$. Note, for **C** and **D**, all libraries were downsampled to be comparable to PBAT (150 million reads, or $\sim$4.8X coverage, per sample, see "Methods" for details); therefore, any differences are not likely confounded by sequencing depth. As expected, this coverage will be substantially higher at increased depth

**Fig. 4:**
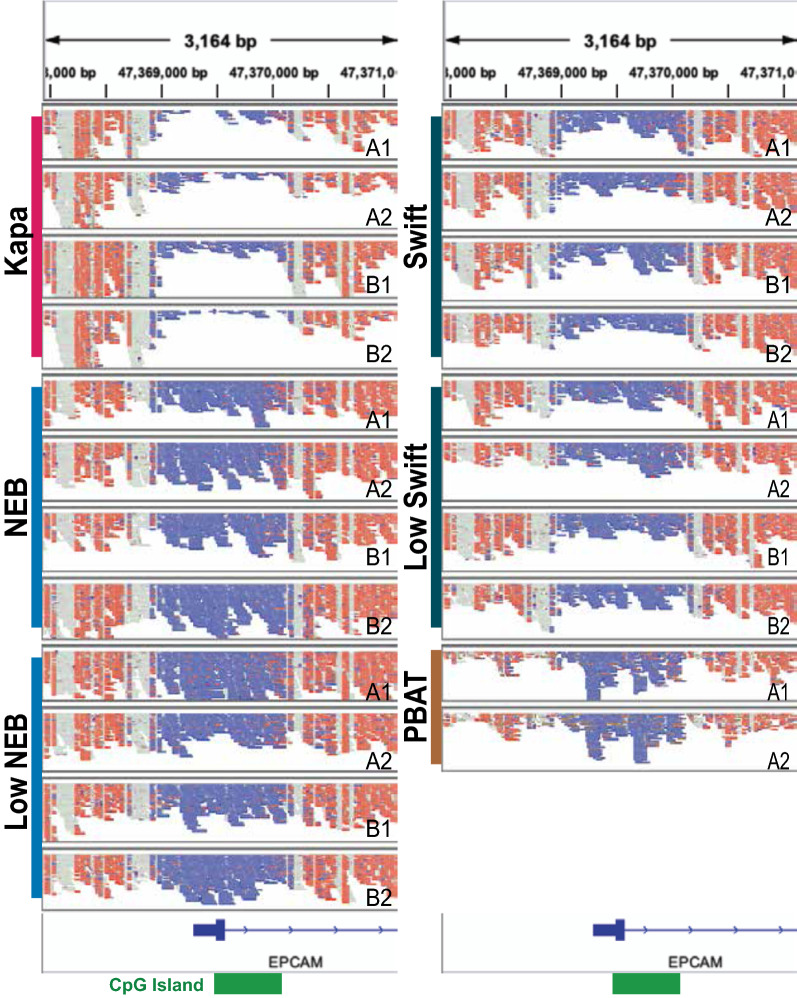
The EPCAM promoter region as a representative example for data generated with the protocols. The aligned reads tracks are taken from the Integrated Genomics Viewer (IGV) [45] in the bisulfite mode where red represents an unconverted cytosine and blue represents a converted cytosine. Each panel represents one sample, with A and B denoting the biological replicates and 1 and 2 the technical replicates for each library construction protocol. The shown region is 1500 bp upstream and downstream of exon 1. The location of a CpG island is indicated with a green box on the bottom. Note, the strands for the PBAT samples have been flipped in silico before being displayed to account for the strand definition in the Miura and Ito protocol. The strands in the PBAT protocol are opposite from what is expected by IGV, as well as the definition used by the other protocols. All libraries were downsampled to be comparable to PBAT; therefore, any differences are not likely confounded by sequencing depth. As expected, this coverage will be substantially higher at increased depth

To determine the uniformity of reads distributed across the genome, the ratio of the observed read coverage to the expected coverage was calculated across several regions (Fig. 3A–B and Additional file 1: Figure S7). In general, there is consistent coverage across the genic, intergenic, and repeat-masked regions, with each sample having less than a 5% departure from expected uniformity (closer to 1.0 is better) in these regions (Additional file 1: Figure S7A–C). In contrast, exonic regions, all CpGs, and CpG islands show greater heterogeneity across kits and larger departures from uniform coverage (Fig. 3A–B and Additional file 1: Figure S7D). PBAT had a much higher observed rate of coverage compared to expected. The other protocols all have lower coverage than would be expected, with the NEB samples having the closest to uniform coverage and the Kapa samples having the lowest coverage of these regions. The undercoverage of CpGs by the Kapa protocol is consistent with a prior study [19]. One example of the differences in coverage uniformity across the protocols can be found in the EPCAM promoter region (Fig. 4).

When performing WGMS, a library preparation’s coverage of cytosines, particularly in a CpG context, is important to assess the DNA methylation landscape of a given sample. When comparing the protocols used in this analysis, the Kapa protocol has the lowest percentage of CpGs covered in a number of different regions on these samples (Fig. 3C–D and Additional file 1: Figure S8), which is consistent with previous work [19]. Generally, the other preparations have coverage over 85% at 150 million mapped reads, with the exception of the Swift samples’ coverage of CpG islands, which is in the 75-80% range. It should be noted that the low DNA input runs of the NEB protocol produced coverage levels that are consistent with the standard DNA input runs across samples and technical replicates.

**Fig. 5:**
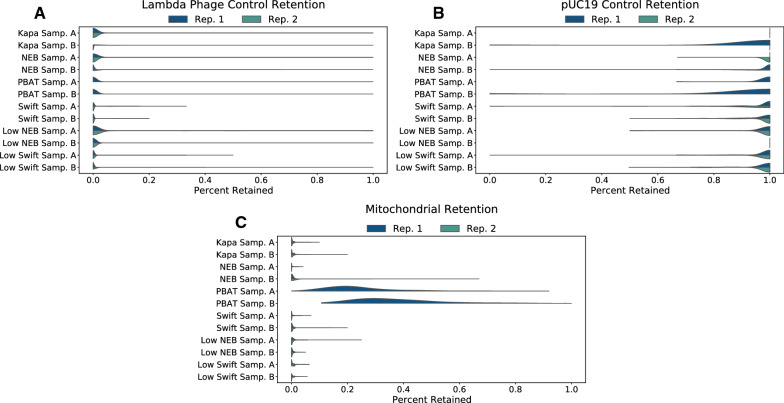
Cytosine retention for methylation controls, namely lambda phage (**A**), pUC19 (**B**), and mitochondrial DNA (**C**). Lambda phage and pUC19 are added to the genomic DNA to serve as unmethylated and methylated controls, respectively. Mitochondrial DNA is a good source of unmethylated DNA that can be used in lieu of spike-in controls. Note, PBAT only had one technical replicate, so there is no “Rep. 2” half on these violins. In addition, the mitochondrial DNA required at least three reads covering each CpG, while the spike-in controls required at least one read due to the fewer number of reads relative to the genomic DNA. This coverage requirement can result in all CpGs for a sample being 1.0, such as in Kapa Sample A in **B**

These methods, bisulfite- or enzyme-based, all distinguish DNA methylation states by converting unmethylated cytosines into uracils and subsequently thymines during PCR, while sparing methylated cytosines. Therefore, effective conversion of unmethylated cytosines, but not methylated cytosines, is key to the accuracy of these methods. Using mitochondrial DNA, which is consistently unmethylated, or spike-in controls, such as unmethylated lambda phage or methylated pUC19 vectors, the effectiveness of the conversion can be tested. Fig. 5 shows the results of cytosine conversion on lambda phage (Fig. 5A), pUC19 (Fig. 5B), and mitochondrial DNA (Fig. 5C). Generally, the cytosine conversion behaved as expected, with the exception of mitochondrial DNA in the PBAT protocol. The beta values show a broad distribution centered slightly below 0.2 for Sample A and about 0.25 for Sample B. Interestingly, the mitochondrial DNA-based incomplete-conversion rate was different from that based on lambda phage for PBAT. This is likely because mitochondrial DNA is circular and can become supercoiled. Unlike the other protocols, there is no mechanical shearing of the DNA in PBAT, which could explain this difference. This also shows the limitation of using mitochondrial DNA as negative controls.

**Fig. 6:**
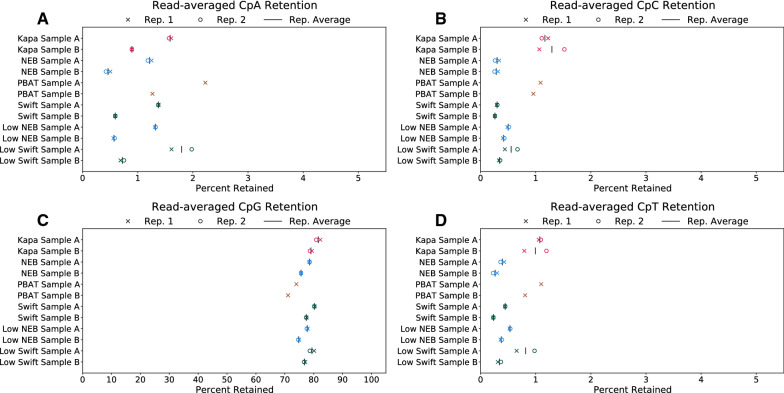
Read-averaged cytosine retention by dinucleotide context:** A** CpA, **B** CpC, **C** CpG, and **D** CpT. In each panel two technical replicates are shown for each biological replicate. The x-axis denotes percent retention, with a scale of 0–5% for CpH panels and 0–100% for the CpG panel. All libraries were downsampled to be comparable to PBAT; therefore, any differences are not likely confounded by sequencing depth

The read-averaged cytosine retention for all protocols reflect what would be expected for a WGMS run, namely CpG retention above 70% and the other cytosine contexts around 1% (Fig. 6). Moreover, the technical replicates showed consistent retention, while the two biological replicates showed a bigger difference. Across all protocols, Sample A consistently had higher CpA retention, but not CpC or CpT retention. It is known that biological CpH retention tends to occur in the CpA dinucleotide context [20]. The higher CpA retention, in contrast to CpC and CpT retention, shows Sample A likely has true CpH methylation, a process previously thought to be largely restricted to embryonic stem cells and neurons [21]. Our results also confirm that, unlike CpA methylation, CpC and CpT retention likely do not reflect true biological methylation, but incomplete conversion, at least in mammalian samples. The Swift and NEB protocols generally showed the lowest amount of such technical artifact (both below 0.5%), except for one replicate of the low input Swift preparation. Similar results can be seen for the base-averaged cytosine retention (Additional file 1: Figure S10).

The M-bias plots [22] (Additional file 1: Figure S11) show a consistently lower CpG retention across the entire read length for both reads 1 and 2 of the PBAT protocol. For the Kapa protocol, there is a consistently higher CpG retention rate for read 1 than the other preparations. However, read 2 tends to be more in line with the retention rate seen in the others. For CpH retention, each protocol has approximately the same level of retention, with slight deviations in the first 5 bp. Due to the adapter trimming performed (see "Methods" for details), retention rates can behave erratically at the end of reads depending on the base content of the adapter that is trimmed. It should also be noted that, by default, the aligner used in this analysis (see "Methods" section) does not include cytosines in the first and last 3 bp of a read when determining methylation beta values, so erratic behavior on the ends of the reads is not included in methylation-related metrics.

**Fig. 7:**
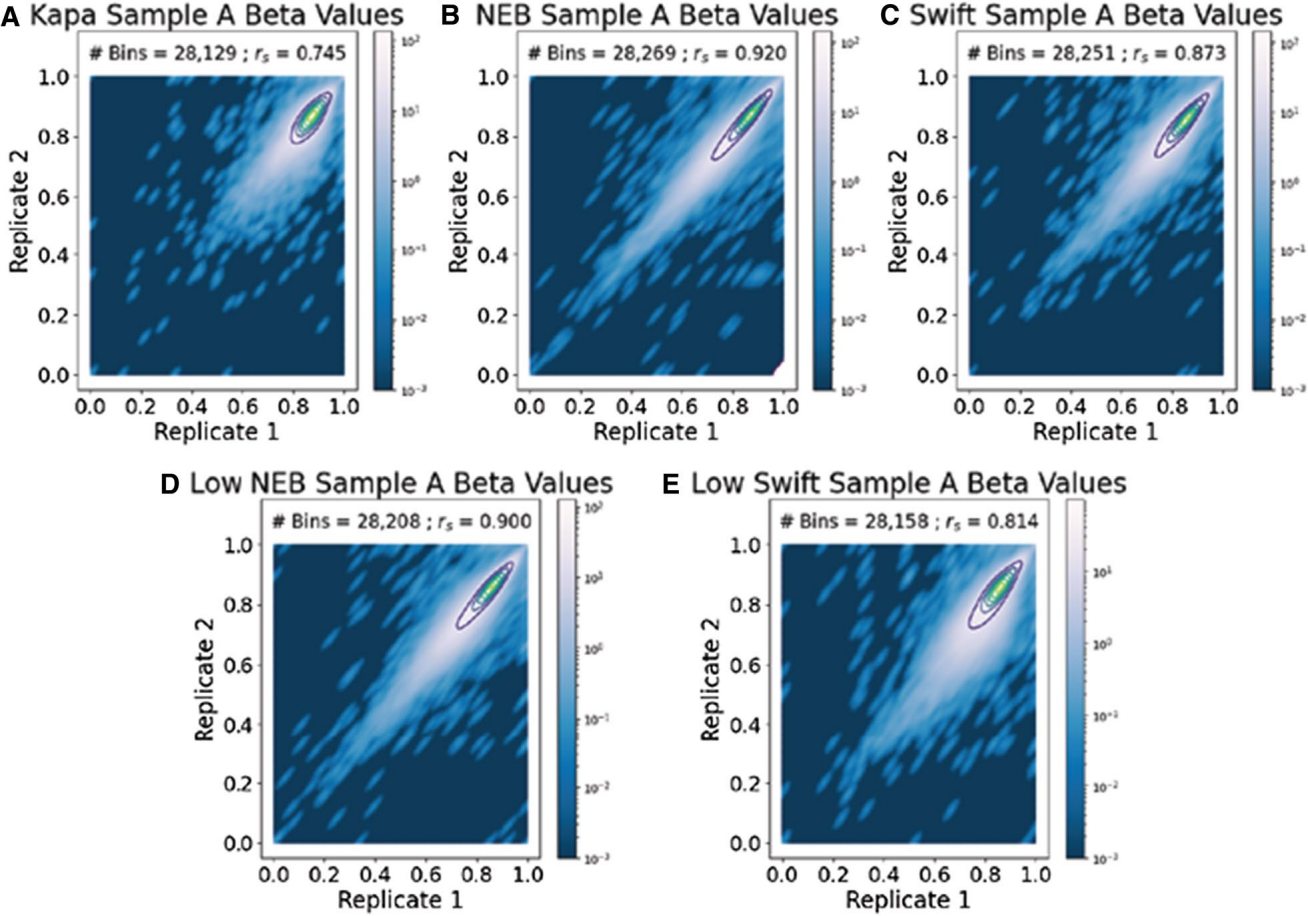
The NEB protocol has the highest correlation of beta values between Sample A technical replicates. The Spearman correlation coefficient, $r_s$, between the two replicates is listed in each figure, along with the number of 100 kb bins used in calculating the coefficient. Note, all libraries were downsampled to be comparable to PBAT; therefore, any differences are not likely confounded by sequencing depth. Overall low correlation values are due to low coverage from downsampling. Additional file 1: Figure S12 shows projections of Replicates 1 and 2 onto a single axis

To compare the consistency of CpG beta values, Spearman correlation coefficients were calculated between technical replicates of each protocol (Fig. 7), as well as between preparations of the same sample (Additional file 1: Table S1). Again, all samples were subsampled to 150 million reads (equivalent of $\sim$4.8X coverage) to ensure fair comparison. The correlation would be higher if the number of reads increases. The correlations between the NEB technical replicates, both the standard and low-input samples, had the highest correlation at 0.9 or higher. The Kapa protocol had the lowest correlation (i.e., less consistency in beta values between replicates) of just under 0.75. The standard-input Swift sample did better than the low-input sample, with correlations of 0.873 and 0.814, respectively. A correlation for the PBAT protocol could not be calculated because PBAT did not have a technical replicate. When comparing preparations to one another, the Kapa protocol had low correlations, with the maximum correlation of 0.62 to the Swift protocol (Additional file 1: Table S1). Only two of the other ten correlations were 0.8 or below, both of which were between the PBAT and Swift protocols. Of the NEB and Swift protocols, the NEB protocol had the highest cross-protocol correlations, with overall greater correlations compared to the Swift protocols.

**Fig. 8:**
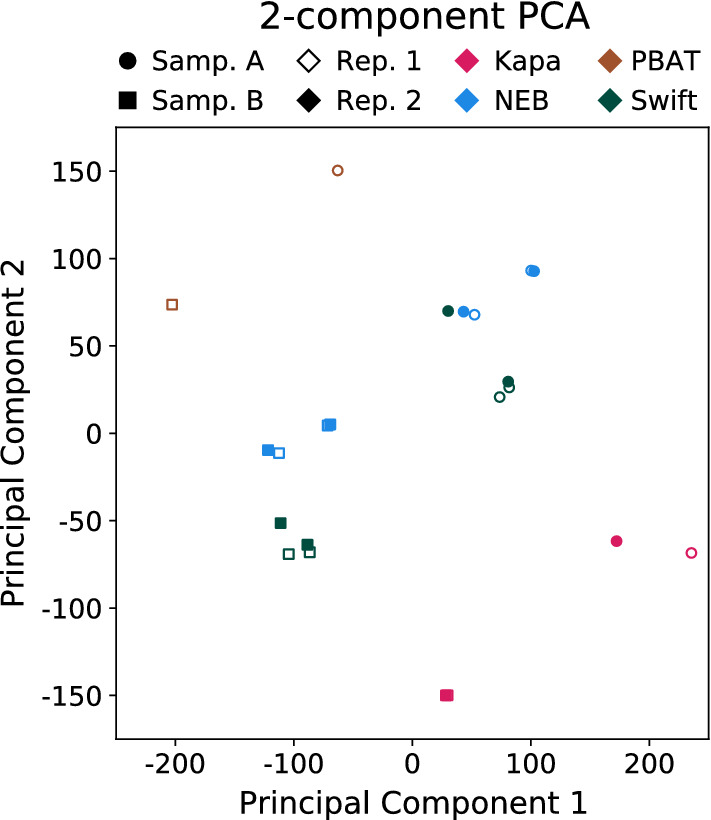
Principal components of average methylation levels in 100 kb bins. Principal component 1 accounts for 41.4% of the variance and principal component 2 accounts for 21.6%. The four protocols are presented with different colors. Biological replicates are displayed with shapes, while technical replicates are shown with different empty or filled markers. With regards to the two Kapa Sample B technical replicates, these points sit almost on top of one another, making them hard to distinguish at the scale shown. Note, all libraries were downsampled to be comparable to PBAT

To further compare the consistency of CpG beta values, a principal component analysis (PCA) was performed (Fig. 8). The PCA shows the data generally splits along two variables: the sample the data came from and the protocol used to generate the library. With respect to the sample split, Sample A clusters in the upper right of the plot, while Sample B clusters in the lower left. The protocol split occurs consistently in both samples, with PBAT and Kapa each separated into their own clusters, while Swift and NEB yielded similar results.

Because of the different approach to cytosine conversion used by the NEB protocol relative to others (enzymatic versus chemical), the difference in beta values between the standard-input NEB and Swift protocols were compared to look for bias in the methylation level of the NEB protocol. After calculating the beta value differences for Sample A, only four CpGs with $|diff| > 0.5$ for both Technical Replicate 1 and 2 were found (Additional file 1: Figure S13). However, in Sample B, only one (which is not one of the four CpGs in Sample A) was seen, so this difference was taken to be sample-dependent and not due to an inherent bias in the enzymatic conversion process.

## Discussion

After comparing the four different WGMS library preparations, we noted that the NEBNext Enzymatic Methyl-seq kit (EM-seq) performed better in almost every metric, both with a standard amount (200 ng) and a low amount (10 ng) of input DNA. The NEB samples had higher quality libraries, with larger insert sizes and higher library complexity than the other kits, while having at least a comparable, if not more uniform, distribution of reads across the genome. The larger insert sizes lead to better mappability during alignment (i.e., a higher fraction of optimally aligned reads). Moreover, it also facilitates read-level analysis, such as epi-polymorphism [23] and allele-specific DNA methylation analyses. The higher library complexity implies more unique information could be gleaned from deeper sequencing of these libraries. Furthermore, the low-input NEB samples showed little reduction in library complexity relative to the standard-input NEB samples. Because of this, it may be possible to push the lower threshold of DNA input below NEB’s recommended limit of 10 ng. When comparing methylation-related metrics, the NEB samples were among the highest in percentage of CpGs covered, with consistent cytosine retention across all reads and a sub-one percent CpH retention level.

Among traditional bisulfite-based methods, the Swift Biosciences Accel-NGS Methyl-Seq DNA library kit performed best, which is consistent with a previous study that compared Swift with two other bisulfite-based protocols, TruSeq and QIAseq [24]. Its results were comparable enough to the NEB protocol, particularly at the standard-input level (100 ng), such that conclusions drawn from both protocols should be fairly consistent. Its performance slightly trailed that of the NEB as the input amount dropped in terms of library complexity, but nonetheless performed well. It is noteworthy that the comparisons in this study were performed on high-quality DNA from snap-frozen samples. For more challenging specimens such as FFPE samples, or non-conventional samples, bisulfite-based methods may outperform enzymatic methods. In addition, Swift and Kapa both use a uracil tolerant polymerase, which increases processivity for deaminated nucleotides present in FFPE and ancient DNA samples. Moreover, the adaptase module in Swift makes it even more effective for FFPE, as it is designed to work with single stranded material. Therefore, it likely can better tolerate nicked strands from the FFPE process (as well as those generated from bisulfite conversion). Indeed, in our preliminary studies, the Swift kit performed best for FFPE samples (data not shown).

All kitted protocols had comparable operability in the lab. Kapa and Swift took roughly 7 h to process, which can fit in a single workday. The NEB enzymatic protocol was a 9-h protocol but had several convenient stopping points noted to easily break processing into 2 days. Actual hands-on time for all three protocols was about 4.5 h. The PBAT protocol was the only technically difficult and time-consuming one, mostly because of the requirement to make and quality-control solutions. This protocol took between 14 and 16 h to complete, about 7 of which were hands on.

One notable difference separating the four DNA methylation library generation methods is library uniformity, particularly in CpG island regions (CGIs). Kapa library coverage was underrepresented and PBAT overrepresented in these regions, while Swift and NEB generated relatively uniform libraries (Figs. 3 and 4). We propose that mechanistic differences in library generation account for this phenomenon, with both the relative timing of bisulfite conversion and the choice of random priming versus adapter ligation affecting the CGI coverage in the downstream dataset. In the Kapa protocol, sheared and adapter-ligated libraries undergo bisulfite conversion, causing preferential strand breakage in CGI regions and inhibiting post-bisulfite conversion PCR steps, as both priming regions are no longer present on the library fragment. In the PBAT protocol, libraries are not sheared. Instead, the bisulfite conversion induces random DNA breaks [25–28], again more prevalently in the CGI regions, making these small fragments more available for continued library generation than larger fragments. In addition, CGIs are G:C rich; during the random priming step of the PBAT library creation process, these higher G:C regions generate more stable primer annealing conditions and increase the likelihood of extension and incorporation into the final library due to a slightly higher regional melting temperature and exhibition of G:C clamping features [29]. The more even CGI coverages exhibited by the Swift and NEB libraries are also protocol related: in the Swift protocol, bisulfite conversion precedes manual shearing and adapter ligation, avoiding the strand-breakage issue and generating relatively diverse libraries, and NEB’s process completely avoids the deleterious bisulfite conversion step through enzymatic reactions.

It should be noted that, while the PBAT protocol favors CpG islands, we do not necessarily suggest using this protocol if one is specifically targeting CpG island regions using sequencing. As they are not PCR amplified, PBAT libraries produce relatively little sequenceable material and, as a result, it was difficult to get enough depth to complete our analysis. This is evident in our lack of replication of the PBAT library, as our technical replicate did not produce enough information for a thorough analysis despite exhausting the library during the sequencing run. Further, as shown here, both NEB and Swift, to a lesser degree, are able to push the input threshold well below that of PBAT, while maintaining better quality libraries and more uniform coverage across the genome.

The clinical utility of DNA methylation has been long recognized, explored, and established, particularly for cell-free DNA (cfDNA)-based early detection of neoplasm. The current gold-standard technologies for DNA methylation profiling are bisulfite-based. Harsh bisulfite treatment is known to cause heavy degradation of the template, which is often scarce to begin with for clinical cfDNA samples. cfMeDIP-seq [30] has been developed to overcome this limitation, pushing the lower input limit to 1–10 ng. However, it is associated with similar limitations of common affinity-based methods [2, 30]. As the enzymatic conversion by the NEB protocol still demonstrates a library complexity at 10 ng comparable to that of regular bulk DNA-based methods, the NEB protocol may prove to be a good alternative approach for clinical early detection work. In addition, with its template-preserving nature and excellent performance at low-input levels, the NEB protocol could hold more promise for single-cell DNA methylation profiling.

## Conclusion

In this study, we compared four commonly used WGMS library preparation protocols, including three bisulfite-based protocols and one enzyme-based protocol. Table 1 shows a summary of the protocols used, while Table 2 summarizes some of the results found in the analysis. We found the NEBNext Enzymatic Methyl-seq and the Accel-NGS Methyl-Seq kits performed quantitatively better than the other two protocols at the standard-input level of DNA for each kit. We found the NEB kit to perform comparably across biological and technical replicates for two different amounts of DNA input, whereas the Swift kit showed some decline with the lower amount of input. Based on these results, we recommend use of the NEBNext Enzymatic Methyl-seq kit for whole-genome DNA methylation sequencing.

**Table 2:** Summary of a subset of results found in this analysis

Short Name	Kapa		NEB		PBAT		Swift		Low NEB		Low Swift	
Sample	A	B	A	B	A	B	A	B	A	B	A	B
# Sequenced Reads (Millions)	347.5	607.9	355.6	389.9	127.9	124.1	395.8	368.2	453.7	408.6	392.7	449.8
Low Quality Bases Trimmed (R1)	2.3	1.8	1.0	1.0	0.9	0.9	0.6	0.6	1.2	1.1	1.1	0.8
Low Quality Bases Trimmed (R2)	2.0	1.7	1.1	1.0	5.9	3.4	0.7	0.7	1.2	1.2	1.0	0.8
Insert Size	208.1	228.0	285.4	297.8	299.3	300.6	222.1	225.0	291.4	290.8	217.9	223.8
Duplicate Rate	27.5	32.4	9.5	9.9	13.5	11.0	12.8	12.1	18.0	16.2	30.7	26.9
% CpGs Covered	74.0	72.3	87.5	87.6	81.8	82.6	85.6	85.2	87.6	87.6	85.5	85.3
% CpG Retention	81.6	79.0	78.5	75.6	74.0	71.1	80.3	77.5	77.8	74.8	79.4	76.9

All values shown are averaged across the two technical replicates, with the exception of PBAT, which only had one technical replicate. The first four rows are taken from the raw data, while the last three rows are taken from the subsampled data, where the BAMs were downsampled to be comparable to PBAT

## Methods

### Fallopian tube sample preparation

Fallopian tubes from two primary patients were delivered in saline from a local hospital. Upon arrival, samples were washed using sterile 1x Phosphate Buffered Saline and snap-frozen in liquid nitrogen. At a later date, the fallopian tubes were thawed and minced. DNA was extracted from the minced fallopian tubes using the tissue protocol from Qiagen’s DNeasy Blood & Tissue Kit (69504). Following extraction, dsDNA was quantified using Invitrogen’s Qubit 3.0 Fluorometer.

### Whole-genome methylation sequencing libraries

Methylated pUC19 and unmethylated lambda phage DNA (0.0005% and 0.01%, respectively) were added to each high molecular weight genomic DNA sample. These are included as methylation controls in the NEB protocol and were added to the DNA used in each protocol for consistency. The DNA was then sheared to approximately 350 bp in average size for all prepared libraries, with the exception of the post-bisulfite adapter tagging (PBAT) libraries, where the DNA was not initially sheared.

Libraries were prepared from the KAPA Hyper Prep kit (v6.17) (Roche Sequencing, Cat. #KK8504) with an input of 300 ng of sheared DNA following the manufacturer’s protocol with the following modifications. Illumina TruSeq Nano adapters at a concentration of 10 $\mu$M were used. The post ligation cleanup elution was reduced to 20 $\mu$L and the entire DNA elution went into the EZ DNA Methylation-Gold kit (Zymo Research, Cat. #D5005). The bisulfite converted DNA was eluted in 20 $\mu$L and 10 cycles of library amplification were performed using the KAPA HiFi HotStart Uracil+ ReadyMix (Roche Sequencing, Cat. #KK2800).

Libraries were prepared from the Accel-NGS Methyl-Seq DNA library kit (v3.0) (Swift Biosciences, Cat. #30024) with an input of either 10 ng or 100 ng of sheared DNA following manufacturer’s protocol with the following modifications. The DNA was bisulfite converted using the EZ DNA Methylation-Gold kit (Zymo Research, Cat. #D5005) with an elution volume of 15 $\mu$L. Following adapter ligation, either 8 cycles (10 ng DNA input) or 4 cycles (100 ng DNA input) of library amplification were performed.

Libraries were prepared from the NEBNext Enzymatic Methyl-seq kit (New England Biolabs, Cat. #E7120L) using an input of either 10 ng or 200 ng of sheared DNA and libraries were made according to the manufacturer’s protocol. The denaturation method used was 0.1 N sodium hydroxide, according to the protocol, and either 8 cycles (10 ng DNA input) or 4 cycles (200 ng DNA input) of PCR amplification were performed.

Libraries were prepared from the PBAT method described by Miura and Ito [13] using an input of 100 ng of genomic DNA that went directly into the EZ DNA Methylation-Gold kit (Zymo Research, Cat. #D5005) with an elution volume of 21 $\mu$L. Then, 10 $\mu$L of the bisulfite converted DNA was used for making the PBAT libraries as previously described in [13] with the modification of using KAPA HiFi HotStart Uracil+ ReadyMix (Roche Sequencing, Cat. #KK2800) for the DNA template extension step.

KAPA pure beads (Roche Sequencing, Cat. #KK8001) were used for cleanup steps for all prepared libraries.

Quality and quantity of the finished libraries were assessed using a combination of the Agilent High Sensitivity DNA chip (Agilent Technologies, Inc., Cat. #5067-4626), QuantiFluor^®^ dsDNA System (Promega Corp., Cat. #E2670), and Kapa Illumina Library Quantification qPCR assay (Roche Sequencing, Cat. #KK4824). 100 bp paired-end sequencing was performed on an Illumina NovaSeq6000 sequencer using an S4, 200 bp sequencing kit (Illumina Inc., San Diego, CA, USA), with 10% PhiX. Base calling was done by Illumina RTA3 and output of NCS was demultiplexed and converted to FASTQ format with Illumina Bcl2fastq (v1.9.0).

### Alignment and methylation extraction

Upon receipt of the FASTQ files, the files were trimmed using Trim Galore [31] version 0.6.4_dev (using Cutadapt version 2.10). Default inputs were used, other than: –illumina –trim-n –paired –cores 4 –fastqc –fastqc_args "–noextract". In addition, the FASTQ files for the Swift samples included –clip_R2 14, due to the Adaptase^™^ method used by Swift Biosciences [32].

The trimmed FASTQ files were aligned to GRCh38 [33] using BISCUIT [34] version 0.3.16. An index for the reference genome was created using biscuit index GRCh38.p13.genome.fa, followed by aligning each sample to the indexed reference. The alignment step used the default options for biscuit align, with these exceptions: -M -t 20 -R sample_specific_read_group. Each sample received its own read group (-R tag). The aligned reads were duplicate marked using Samblaster [35] version 0.1.25, with the -M flag and defaults. The reads were then coordinate sorted and indexed using Samtools [36] version 1.10 (with htslib 1.10.2). Default options were used, with the following exceptions -@ 20 -m 5G -o sample_name.sorted.markdup.bam -O BAM (sort) and -@ 20 (index).

The extraction of cytosine methylation information proceeded as follows. Pileup VCF files were generated using biscuit pileup with default parameters. bgzip and tabix (included with htslib version 1.10.2) were used to compress and index the VCF files. Default parameters were used for bgzip and tabix, with the exception of -p vcf in the call for tabix. The VCF files were then processed through biscuit vcf2bed, bedtools sort (bedtools [37] version 2.29.2), and biscuit mergecg to create coordinate sorted BED files containing CpG methylation beta value information. For each command, the default parameters were used. After creating the BED files, they were compressed and indexed using bgzip and tabix, with default parameters being used in both cases, with tabix also including -p bed.

Any code not explicitly stated in this and subsequent sections can be found on GitHub [38].

### FASTQ and alignment quality control

Quality-control data were collected from a number of sources and viewed using MultiQC [39] version 1.9. Quality control for the FASTQ files was generated by FastQC [40] version 0.11.9 and Cutadapt during the trimming process. Command arguments used can be found in the previous section. Statistics on the percentage of duplicate marked reads were produced by Samblaster. Library complexity was calculated by Preseq [41] version 2.0.3 using these options, c_curve -B -P -v -o sample.complex.ccurve.txt, where “sample” is the name of each sample. Quality controls from Samtools were generated with samtools stats and samtools flagstat, with default parameters and -@ 20. BISCUIT includes a custom BASH script to generate quality-control statistics related to data aligned by BISCUIT. This script was run with this command, QC.sh -v samp.pileup.vcf.gz -o samp_QC hg38_assets GRCh38.p13.genome.fa samp samp.sorted.markdup.bam. In each case, “samp” corresponds to the name of the processed sample.

The hg38_assets mentioned in the BISCUIT quality-control script command can be found in a zip file on the BISCUIT GitHub release page [42].

### Library protocol comparison analysis

To collect statistics related to the raw reads stored in the FASTQ files, a custom Python (version 3.7.6) script was written. It uses the gzip, glob, and time Python base modules and these additional Python packages: argparse (version 1.1), numpy (version 1.18.1), and biopython (version 1.76). Statistics regarding the trimmed reads were collected from log files generated by Cutadapt, as described previously.

For a number of the analyses, the aligned BAM files were subsampled before calculating the corresponding metric. The BAMs were subsampled using samtools view -hbu -F 0x4 -q 40 sample.bam | samtools view -hbu -s FRAC -. “FRAC” was calculated as

1 $$\begin{aligned} \frac{150,000,000}{Number~Mapped~Fragments~with~MAPQ\ge 40}, \end{aligned}$$

for each sample. The subsampled BAMs were sorted and indexed using Samtools. Pileup VCF files and merged CpG BED files were generated in a similar manner to the original BAMs, as described previously.

Using the subsampled BAMs, the average coverage and percentage of covered CpGs within different genomic regions, including CpG islands, exons, genes, and repeat-masked regions, were calculated using custom scripts. The CpGs that fell in each region were determined by intersecting a BED file containing CpG coordinates and the coverage at those locations with a BED file containing the region’s coordinates. The coverage was determined using bedtools genomecov, while the intersection was done using bedtools intersect. The average coverage was calculated by taking a weighted average of the coverage for each CpG. The percentage of covered CpGs was calculated by

2 $$\begin{aligned} \frac{Number~of~CpGs~in~Region~with~Coverage>1}{Total~Number~of~CpGs~in~Region}. \end{aligned}$$

The scripts used to calculate these values made use of GNU parallel [43].

The observed coverage to expected coverage ratio was calculated as $ratio = Obs / Exp$, where:

3 $$\begin{aligned} Obs&= \frac{Number~of~Bases~Mapped~to~Region}{Total~Number~of~Mapped~Bases} \end{aligned}$$

4 $$\begin{aligned} Exp&= \frac{Sum~of~Mappability~Scores~in~Region}{Total~Sum~of~Mappability~Scores~in~Genome}. \end{aligned}$$

This formulation assumes all bases are not equally accessible when sequencing. The expected coverage takes into account this difference in accessibility by including mappability scores based on the Bismap k100 multi-read mappability scores [44]. The observed coverage does not include these scores, as mappability is assumed to be inherently included when performing DNA sequencing. Because Bismap did not include a mappability score for every base in the genome, the expected and observed coverage calculations were restricted to those bases that included a mappability score. Only mapped reads (FLAG field in BAM does not include 0x4 flag) with MAPQ score $\ge 40$ were included in this calculation. The values for each observed to expected ratio were calculated using custom BASH scripts that used bedtools and GNU awk (version 4.0.2).

The beta values for the lambda phage and pUC19 methylation controls were extracted using the same method as the genomic methylation extraction (see Alignment and Methylation Extraction for the details), with the one exception being that only one read was required to cover a CpG. The coverage requirement difference was due to the fractional amount of lambda phage and pUC19 that were included in the sequencing compared with the amount of genomic DNA. The mitochondrial DNA beta values were taken directly from the genomic methylation BED file.

To calculate correlations between samples, the genome was binned into 100 kb bins and the average beta value calculated for CpGs in each window. The bins were determined via bedtools makewindows -w 100000 -g GRCh38.p13.genome.fa.fai | sort -k1,1 -k2,2n. The “.fai” file was generated via samtools faidx GRCh38.p13.genome.fa. The average beta value for each bin was calculated via bedtools map -a bins.bed -b sample.bed -c 4,5 -o mean | gzip. bins.bed contains the 100 kb bins, while sample.bed is the merged CpG BED file generated from the subsampled BAMs. Correlations between the 100 kb averaged beta values were calculated using the Spearman correlation coefficient, as implemented in Python’s scipy package (version 1.4.1).

The PCA was performed using the average beta values in 100 kb bins, which were calculated in the same way as the correlation analysis. After calculating the beta values, they were transformed into smoothed M-values via:

5 $$\begin{aligned} logit \left( \frac{M+k}{(M+k) + (U+k)} \right) , \end{aligned}$$

where *M* is the number of methylated cytosines and *U* is the number of unmethylated cytosines at a given CpG. The smoothing factor, *k*, eliminates the infinities that occur at 0 and 1 in the logit transformation. The numpy logit function was used to apply the transformation. This conversion turns the beta-distributed beta values into the more Gaussian-distributed M-values. After converting to M-values, the data was standardized using the StandardScaler function, as implemented in scikit-learn (sklearn version 0.24.0). The PCA was performed using scikit-learn’s PCA function, keeping only the first two components (n_components=2).

The analysis for methylation bias in the NEBNext Enzymatic Methyl-seq kit was performed by extracting CpGs that had more than 20 reads covering them for both the NEB and Swift protocols for Sample A replicate 1 and 2 or the NEB and Swift protocols for Sample B replicate 1 and 2. Methylation bias was determined by requiring both Equations 6 and 7 to be true.

6 $$\begin{aligned} |\beta _{NEB} - \beta _{Swift}|&> 0.5~(Replicate~1) \end{aligned}$$

7 $$\begin{aligned} |\beta _{NEB} - \beta _{Swift}|&> 0.5~(Replicate~2) \end{aligned}$$

This was done separately for the standard DNA input NEB and Swift protocols of Samples A and B.

The CpG and other genomic region BED files mentioned in this section can be found on the GitHub release page for the analysis code.

## Supplementary Information

**Additional file 1.** Additional Table S1 and Figures S1–S13.

## Data Availability

Sequencing data are from human tissue and will be available through dbGaP. Software used in processing the data is available on GitHub at https://github.com/jamorrison/wgms_kit_comparison/releases/tag/v1.0.4.

## Abbreviations

DNA: Deoxyribonucleic acid
PCR: Polymerase chain reaction
FFPE: Formalin-fixed, paraffin-embedded
WGBS: Whole-genome bisulfite sequencing
EM-seq: Enzymatic methyl-seq
WGMS: Whole-genome (DNA) methylation sequencing
PBAT: Post-bisulfite adapter tagging
C: Cytosine
T: Thymine
mC: Methylated cytosine
5-mC: 5-Methylcytosine
5-hmC: 5-Hydroxymethylcytosine
5-fC: 5-Formylcytosine
5-caC: 5-Carboxylcytosine
TET: Ten–eleven translocation
APOBEC3A: Apolipoprotein B mRNA editing enzyme, catalytic polypeptide-like 3A
