# Supplementary material for: Evaluation of whole-genome DNA methylation sequencing library preparation protocols
Source: Epigenetics Chromatin. 2021 Jun 19;14:28. doi: 10.1186/s13072-021-00401-y (PMC8214260; doi:10.1186/s13072-021-00401-y)
Supplement: Supplementary file 1 — Additional file 1. Additional Table S1 and Figures S1–S13. [file 13072_2021_401_MOESM1_ESM.pdf]

Additonal file 1

	Kapa	NEB	PBAT	Swift	Low NEB	Low Swift
Kapa	–	0.55	0.41	0.62	0.55	0.61
NEB	–	–	0.88	0.87	0.91	0.83
PBAT	–	–	–	0.80	0.86	0.76
Swift	–	–	–	–	0.87	0.83
Low NEB	–	–	–	–	–	0.83
Low Swift	–	–	–	–	–	–

Supplemental Table S1: **Spearman correlation coefficients between protocols for Sample A Technical Replicate 1.** Note, all libraries were downsampled to be comparable to PBAT; therefore, any differences are not likely confounded by sequencing depth. Overall low correlation values are due to low coverage from downsampling.

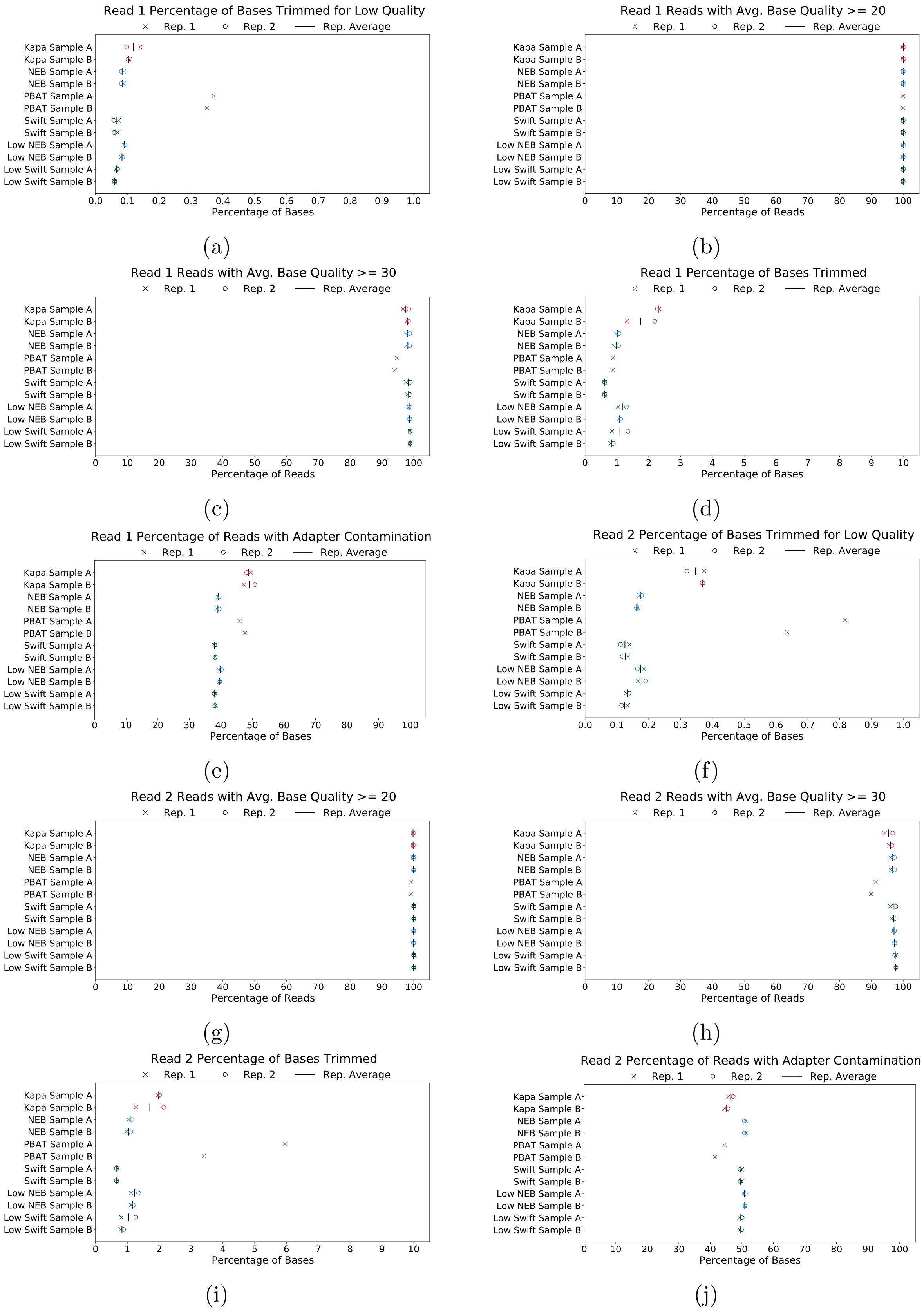

Supplemental Figure S1: **Raw read statistics for each protocol. (a)–(e)** Statistics for read 1. **(f)–(j)** Statistics for read 2.

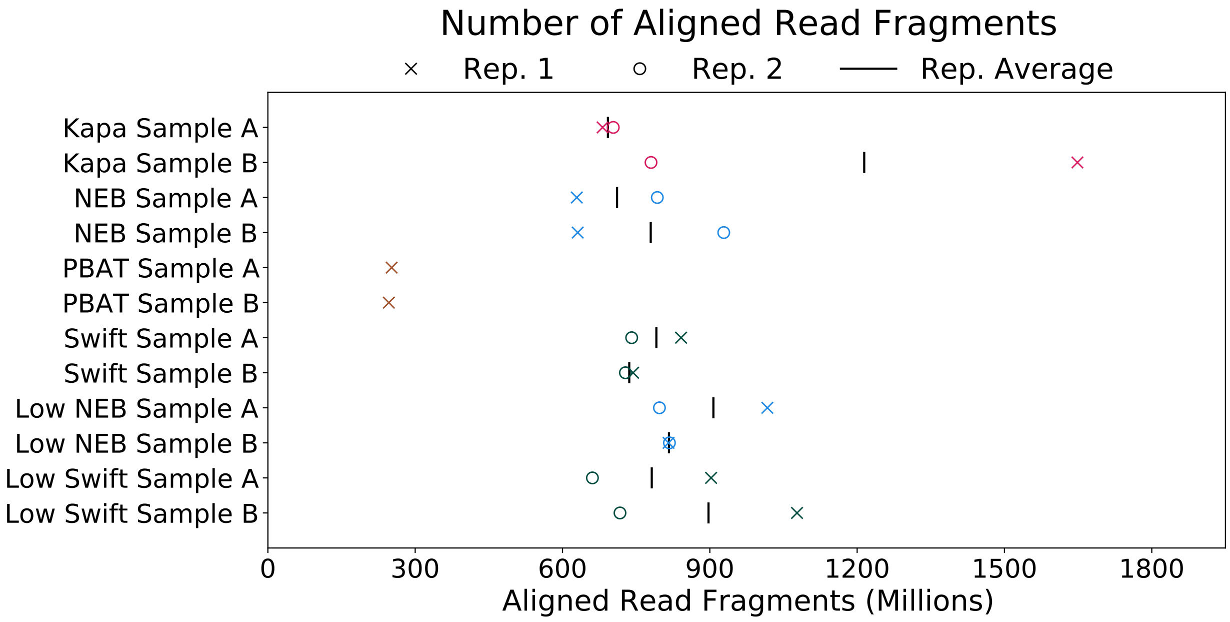

Supplemental Figure S2: **The number of aligned read fragments for each sample.** The number of read fragments treats reads 1 and 2 as distinct entities.

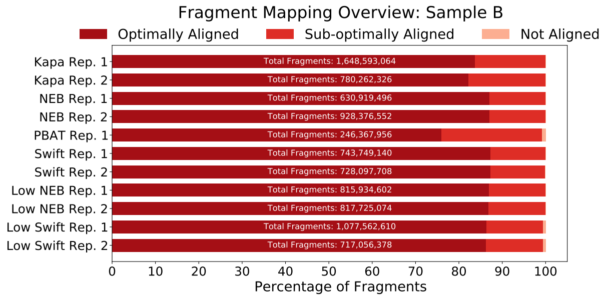

(a)

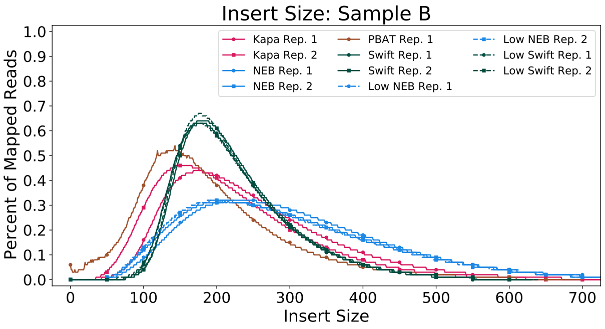

(b)

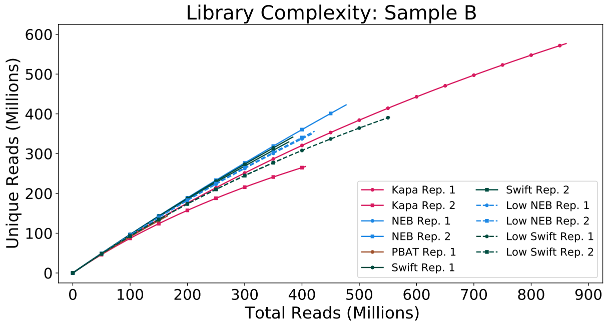

(c)

Supplemental Figure S3: **Library quality metrics for each protocol for Sample B. (a)** The percentage of optimally, sub-optimally, and not aligned read fragments for each protocol. Note, read fragments treat reads 1 and 2 as separate entities, as it is possible that one read in the pair is mapped, while the other is not. **(b)** Insert size distribution. **(c)** The library complexity, which is a function of the duplicate rate.

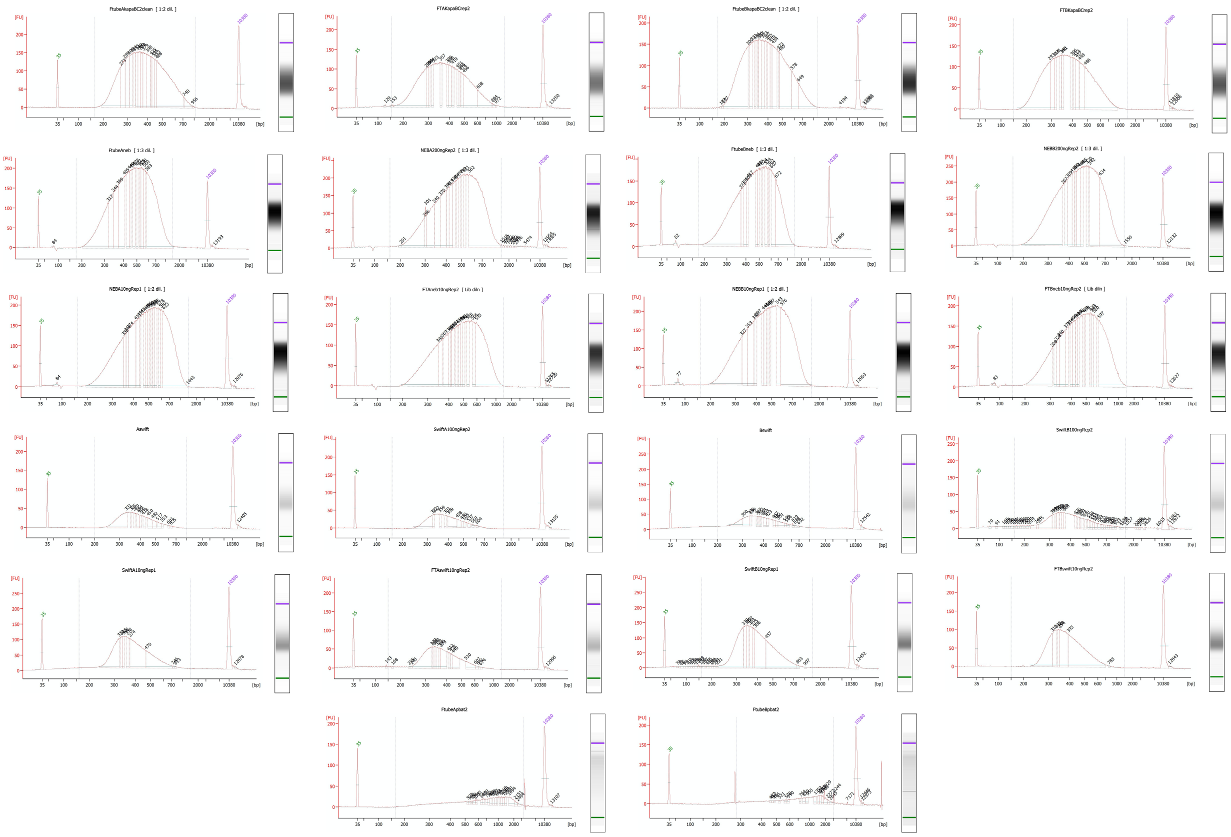

Supplemental Figure S4: **Bioanalyzer traces are shown for each sample.**

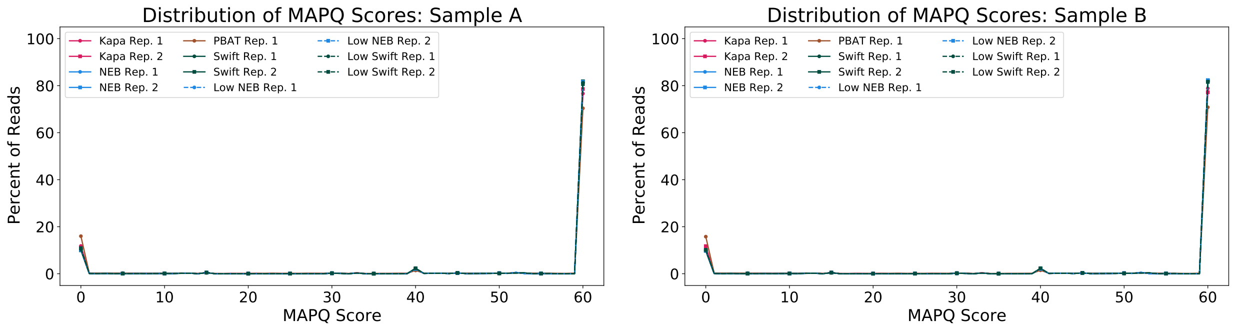

(a) (b)

Supplemental Figure S5: **The distribution of MAPQ scores for Sample A and Sample B.**

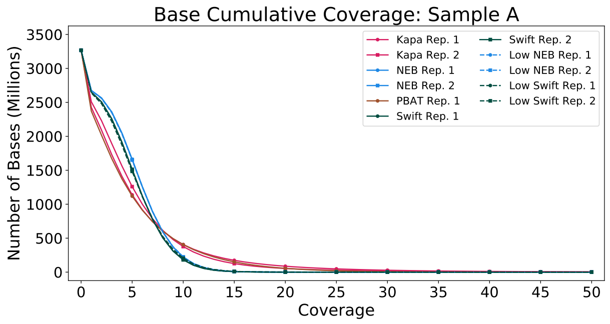

(a)

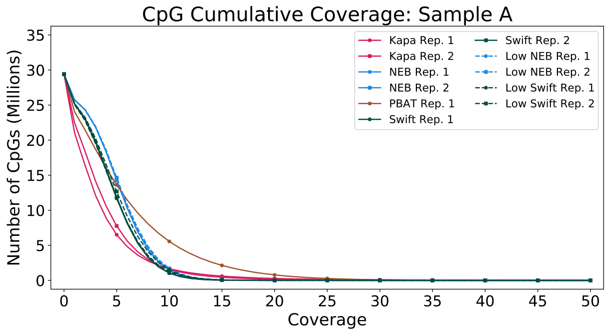

(b)

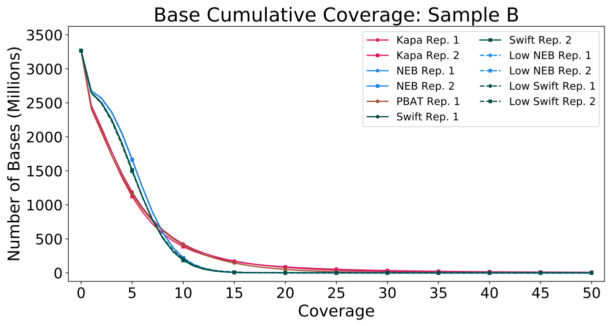

(c)

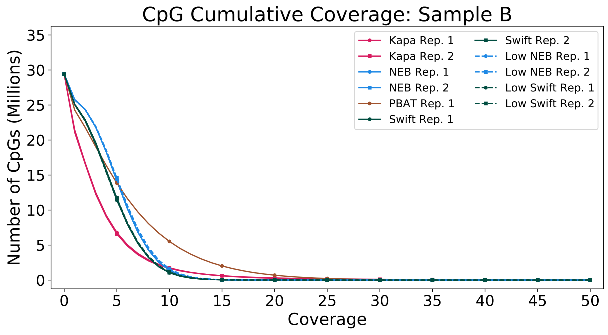

(d)

Supplemental Figure S6: **The cumulative coverage across CpGs and all base for reads with MAPQ $\geq$ 40. (a)–(b)** Cumulative coverage for Sample A. **(c)–(d)** Cumulative coverage for Sample B. Note, all libraries were downsampled to be comparable to PBAT (150 million reads, see Methods for details); therefore, any differences are not confounded by sequencing depth.

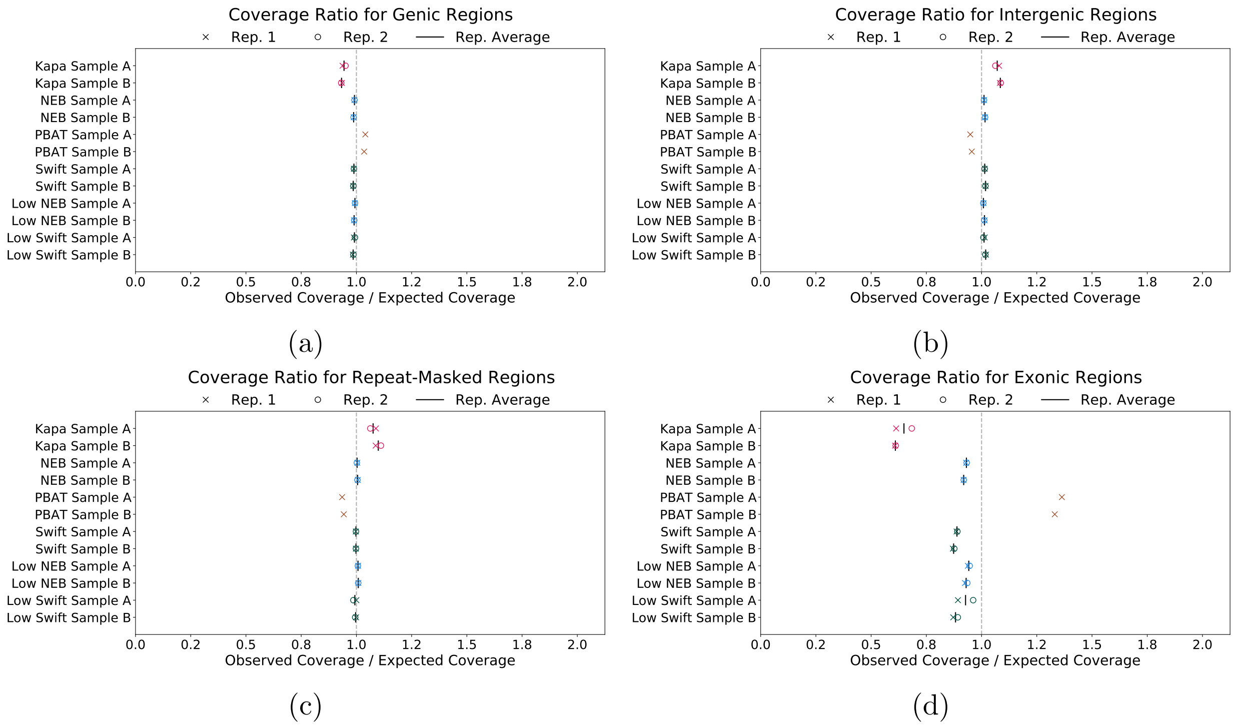

Supplemental Figure S7: **The ratio of observed coverage to expected coverage for various genomic element categories. (a)** Ratio for genic regions. **(b)** Ratio for intergenic regions. **(c)** Ratio for repeat-masked regions. **(d)** Ratio for exonic regions.

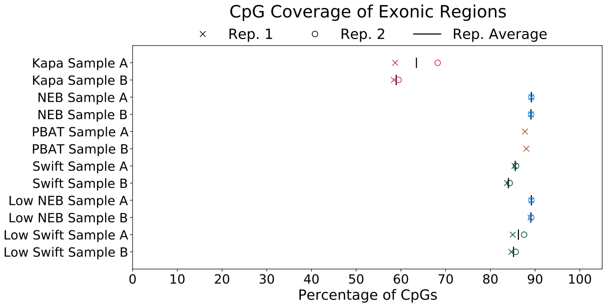

(a)

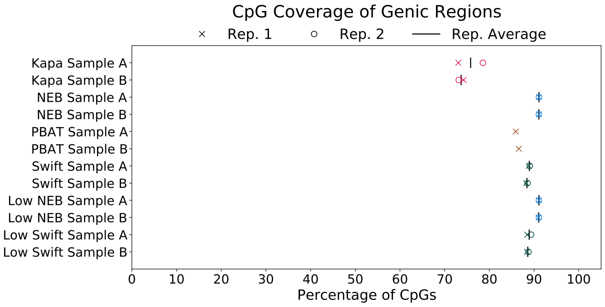

(b)

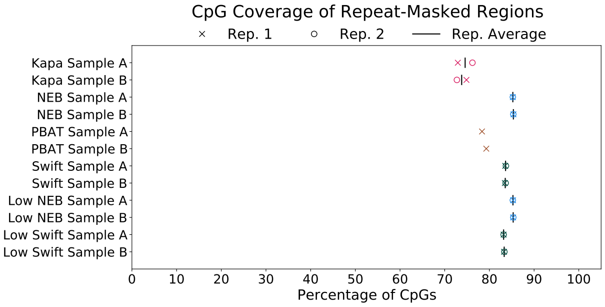

(c)

Supplemental Figure S8: **Percentage of CpGs covered by at least one unique read with MAPQ ≥ 40 for various genomic element categories. (a)** Percentage of covered CpGs in exonic regions. **(b)** Percentage of covered CpGs in genic regions. **(c)** Percentage of covered CpGs in repeat-masked regions. Note, all libraries were downsampled to be comparable to PBAT; therefore, any differences are not likely confounded by sequencing depth.

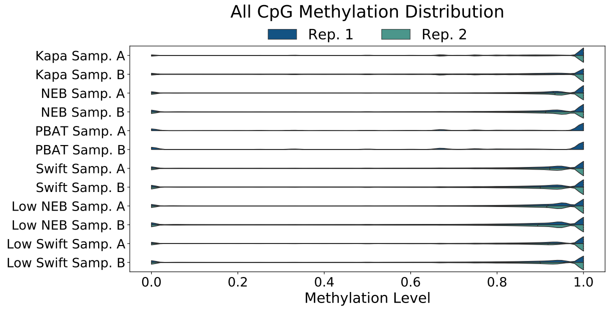

(a)

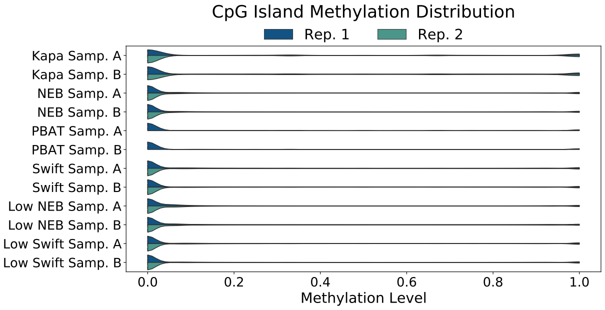

(b)

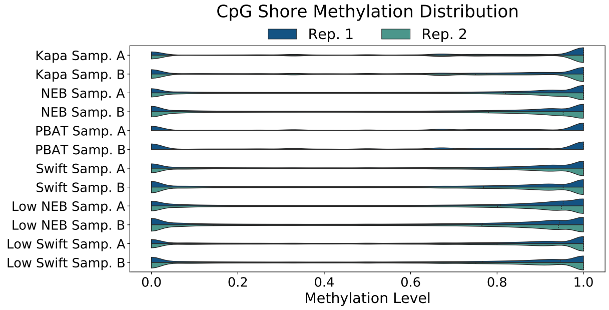

(c)

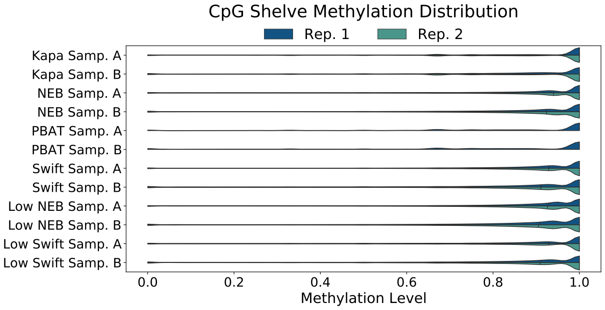

(d)

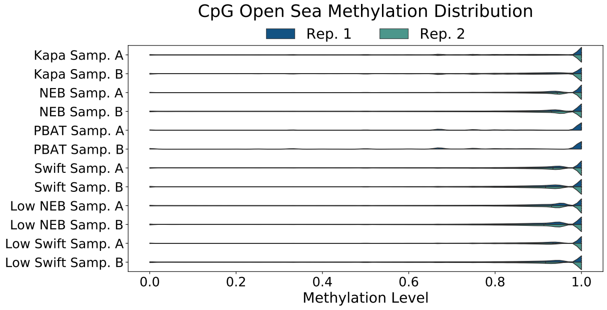

(e)

Supplemental Figure S9: **The distribution of CpG beta values for different genomic regions, including all CpGs (a), CpG islands (b), CpG shores (c, +/- 2000bp from CpG island regions), CpG shelves (d, +/- 2000bp from CpG shore regions), and open seas (e, regions between CpG shores).**

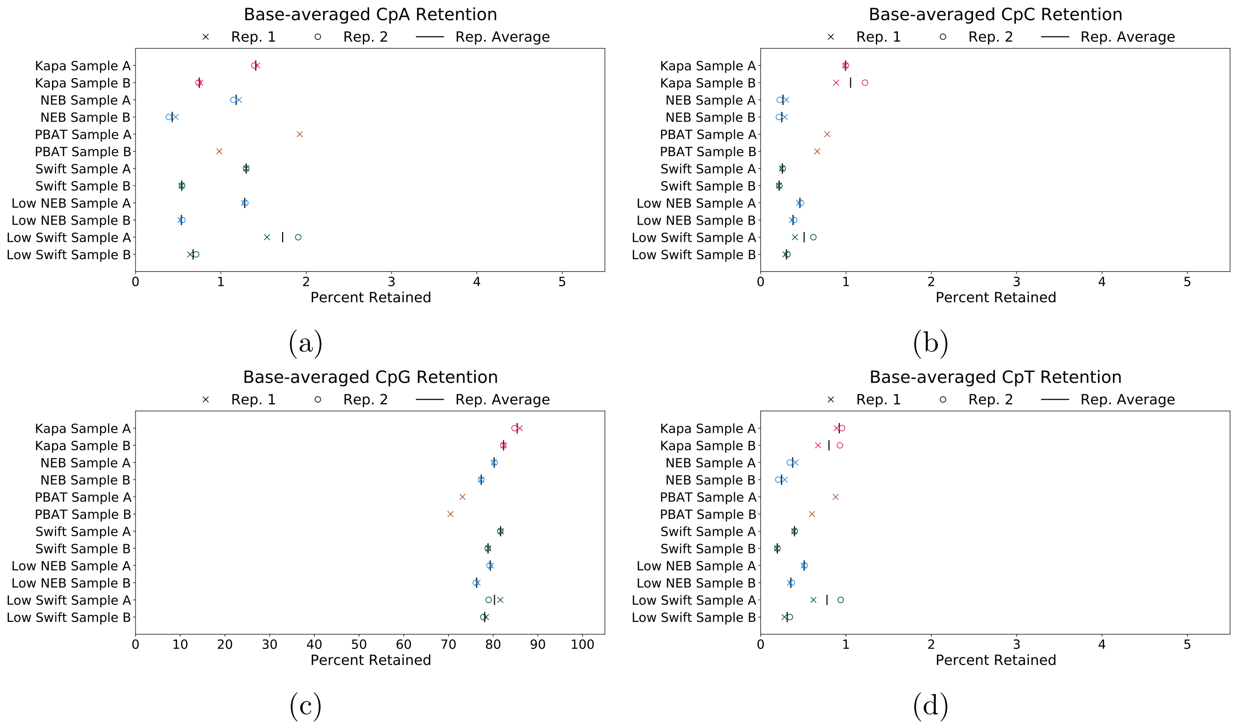

Supplemental Figure S10: **Base-averaged cytosine retention by dinucleotide context, namely (a) CpA, (b) CpC, (c) CpG, and (d) CpT.** In each panel two technical replicates are shown for each biological replicate. The x-axis denotes percent retention, with a scale of 0-5% for CpH panels and 0-100% for the CpG panel.

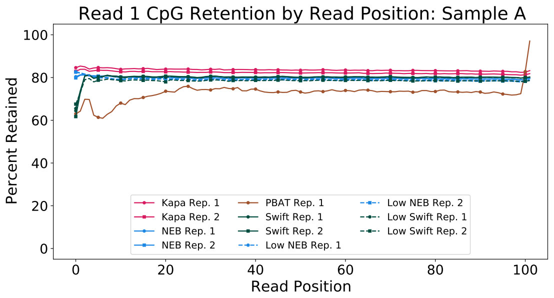

(a)

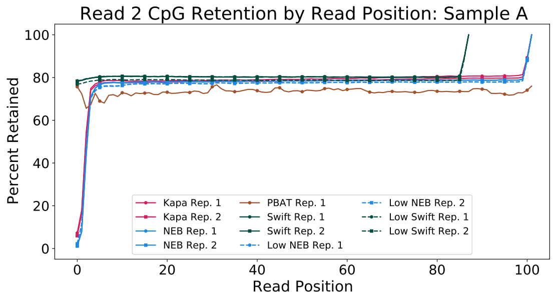

(b)

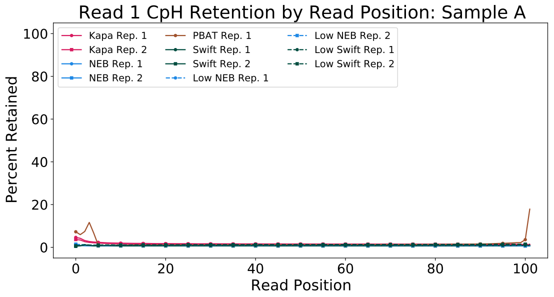

(c)

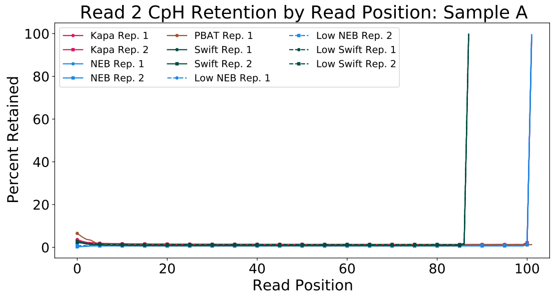

(d)

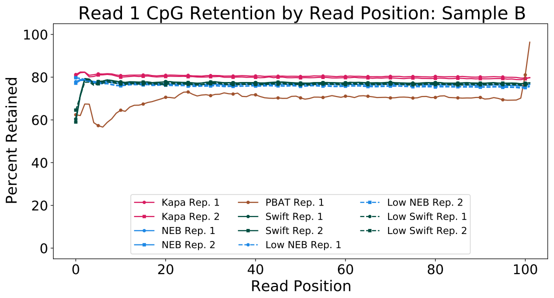

(e)

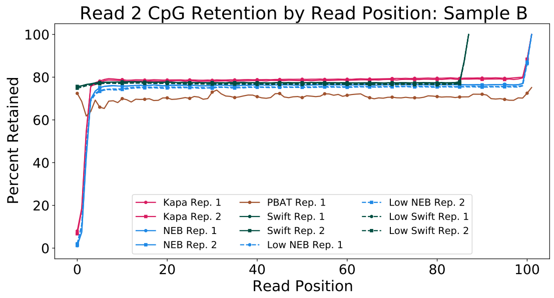

(f)

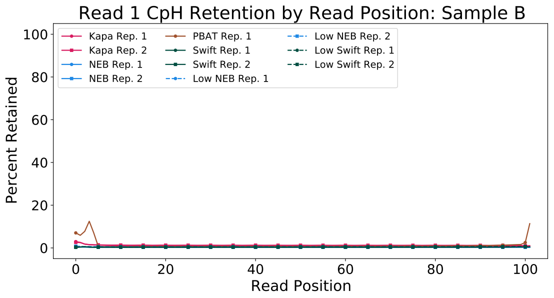

(g)

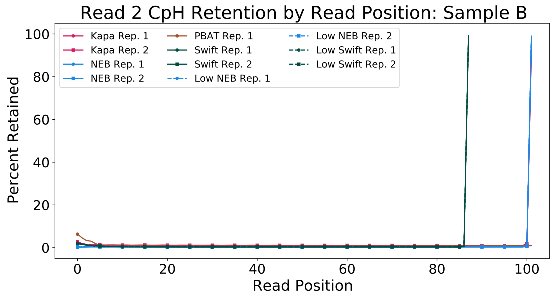

(h)

Supplemental Figure S11: **CpG and CpH retention by read position for each biological sample.** **(a)–(b)** The CpG retention by read position in Sample A. **(c)–(d)** The CpH retention by read position in Sample A. **(e)–(f)** The CpG retention by read position in Sample B. **(g)–(h)** The CpH retention by read position in Sample B. The length of read 2 in the Swift samples is shorter due to additional trimming required for these libraries (see Methods section for details). Note, all libraries were downsampled to be comparable to PBAT; therefore, any differences are not likely confounded by sequencing depth.

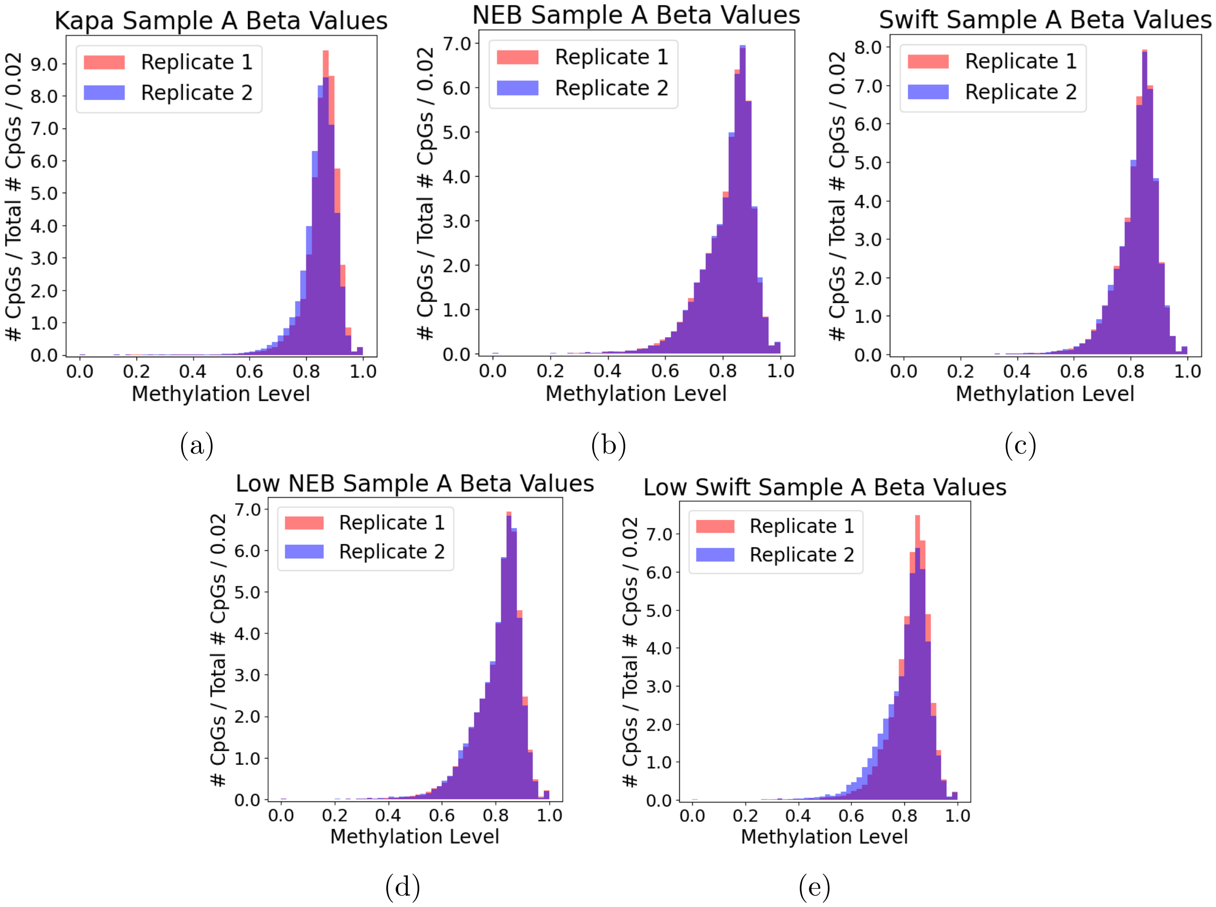

Supplemental Figure S12: **The distribution of CpG beta values for replicates 1 and 2. (a)–(e)** represent the data shown in Figure 7 collapsed onto a single axis.

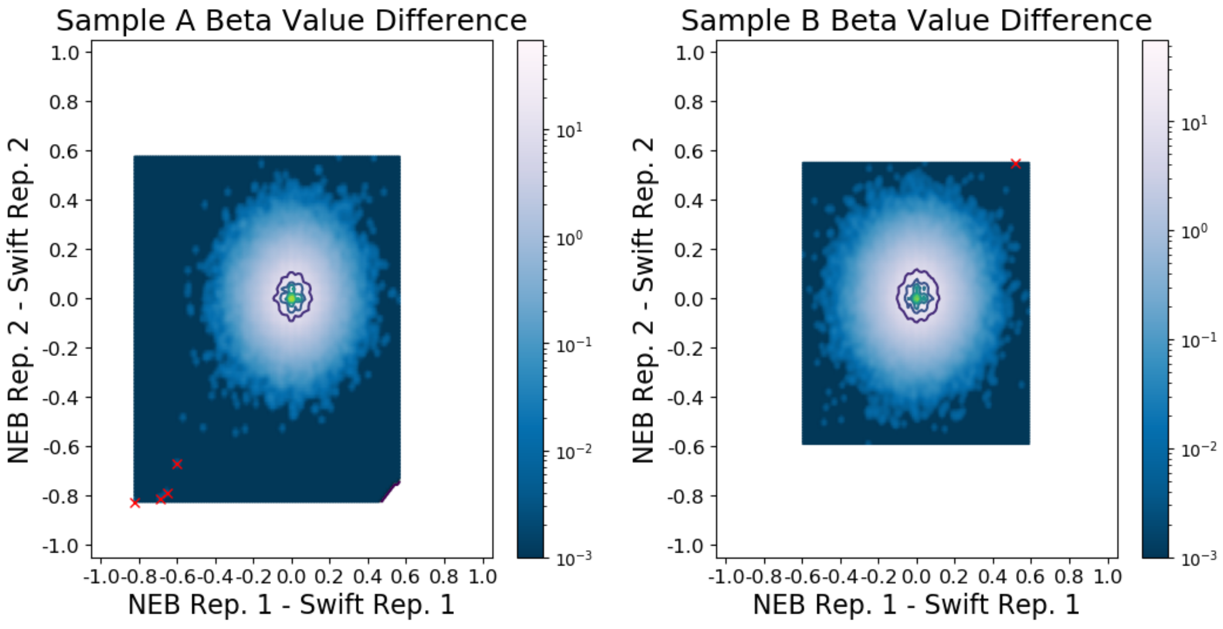

(a)

(b)

Supplemental Figure S13: **Difference in beta values between the NEB and Swift protocols.** Red x's denote CpGs where the difference for both technical replicates exceeds 0.5.
